# Perioperative Anesthetic Management for Bariatric Surgery in a Patient With Achondroplasia and Prior Limb-Lengthening: A Case Report

**DOI:** 10.7759/cureus.106887

**Published:** 2026-04-12

**Authors:** Ji Wook Kim, Dongyul Lee, Donghee Kang

**Affiliations:** 1 Department of Anesthesiology and Pain Medicine, Kosin University College of Medicine, Busan, KOR

**Keywords:** achondroplasia, airway management, bariatric surgery, intratracheal intubation, morbid obesities, oxygen inhalation trherapy, sleep apnea

## Abstract

Achondroplasia presents significant anesthetic challenges that may be amplified by morbid obesity. A 29-year-old woman with achondroplasia and morbid obesity (body mass index 69.0 kg/m²) underwent bariatric surgery after prior lower-limb lengthening of 17 cm. Despite her increased height, she retained achondroplasia-related torso proportions and airway features. Standard ramped positioning was ineffective because of her disproportionately short torso. High-flow nasal oxygen was used to maintain oxygenation during prolonged airway management. After failed intubation with a standard videolaryngoscope blade, the airway was secured using a hyperangulated X-blade. Quantitative neuromuscular monitoring and sugammadex facilitated safe extubation. This case suggests that increased height after limb lengthening should not be assumed to indicate lower upper-airway risk in patients with achondroplasia. Perioperative planning should emphasize body proportions rather than standing height, and high-flow nasal oxygen and hyperangulated videolaryngoscopy may be useful airway strategies.

## Introduction

Achondroplasia is a rare skeletal dysplasia and the most common cause of disproportionate short stature, with an estimated incidence of approximately 0.3-1.0 per 10,000 live births, and poses unique anesthetic challenges, particularly with respect to airway/respiratory care and patient positioning [1,2]. Characteristic craniofacial and upper airway features, including midface hypoplasia, macrocephaly, short neck, apparent mandibular prominence, and a high-arched palate, may complicate mask ventilation, laryngoscopy, and tracheal intubation. Given the possibility of foramen magnum stenosis or cervicomedullary compression, excessive cervical manipulation should be avoided [3]. In addition, kyphoscoliosis, thoracic cage deformity, and coexisting morbid obesity may further impair respiratory mechanics, increase the risk of rapid desaturation during induction, and amplify perioperative risk in the presence of obstructive sleep apnea and/or obesity hypoventilation syndrome [2,3]. In patients with prior limb-lengthening, altered body proportions may further complicate positioning and airway management, although this issue has been rarely emphasized in the anesthetic literature.

Previous reports have highlighted the importance of meticulous airway planning in achondroplasia [4,5]. Here, we describe an adult with achondroplasia who had undergone two limb-lengthening procedures in childhood and later presented for bariatric surgery with a body mass index (BMI) of 69 kg/m². Because altered body proportions after limb lengthening limited the application of a standard ramped position, we describe a perioperative airway strategy incorporating high-flow nasal oxygen (HFNO) for oxygenation optimization and video laryngoscopy for tracheal intubation.

## Case presentation

A 29-year-old woman with severe obesity (BMI 69.0 kg/m²; height 142.3 cm, weight 139.7 kg) underwent laparoscopic sleeve gastrectomy under general anesthesia. This study was approved by the Institutional Review Board (IRB) of Kosin University Gospel Hospital (IRB No. KUGH 2026-03-001), which waived the requirement for informed consent. She was classified as ASA physical status III. The patient was diagnosed with achondroplasia shortly after birth and underwent two limb-lengthening procedures at approximately 10 and 12 years of age, achieving a total height gain of 17 cm. Although her final height was 142 cm, this increase was achieved through lower-extremity lengthening; consequently, her thoracic proportions and upper-airway features were still considered consistent with achondroplasia. She reported a weight gain of >30 kg over the preceding year after the COVID-19 pandemic, with limited ambulation due to knee pain.

Preoperative evaluation revealed profound obstructive sleep apnea with severe oxygen desaturation on polysomnography (apnea-hypopnea index [AHI] 111.9 events/h), along with reduced forced vital capacity, suggesting a possible restrictive ventilatory pattern and limited respiratory reserve (Table 1). An exercise stress test was terminated early because of dyspnea, achieving only 4.5 METs. Preoperative airway assessment revealed a Mallampati class IV view and full dentition. Mouth opening and neck mobility, including cervical extension, were preserved. Characteristic craniofacial features included midface hypoplasia, macrocephaly, short neck, and apparent mandibular prominence.

**Table 1:** Summary of preoperative laboratory and diagnostic findings Values are presented as preoperative findings. Spirometry values are shown as pre- and post-bronchodilator measurements, with percentages indicating predicted values. Normal ranges are provided only for parameters with standardized laboratory reference intervals; dashes indicate that a uniform reference range was not applicable. WBC: white blood cell count; PT: prothrombin time; INR: international normalized ratio; aPTT: activated partial thromboplastin time; AST: aspartate aminotransferase; ALT: alanine aminotransferase; BUN: blood urea nitrogen; hsTroponin-I: high-sensitivity troponin I; NT-proBNP: N-terminal pro-B-type natriuretic peptide; HbA1c: hemoglobin A1c; NGSP: National Glycohemoglobin Standardization Program; ABGA: arterial blood gas analysis; PaCO₂: arterial partial pressure of carbon dioxide; PaO₂: arterial partial pressure of oxygen; HCO₃⁻: bicarbonate; SaO₂: arterial oxygen saturation; ECG: electrocardiography; QTc: corrected QT interval; FVC: forced vital capacity; FEV₁: forced expiratory volume in 1 s; FEF₂₅₋₇₅: forced expiratory flow at 25%–75% of FVC; METs: metabolic equivalents; HR: heart rate; BP: blood pressure; PSG: polysomnography; AHI: apnea-hypopnea index; RDI: respiratory disturbance index; SpO₂: peripheral oxygen saturation; REM: rapid eye movement sleep; TST: total sleep time.

Category	Parameter	Result	Normal range
Laboratory	WBC	13.19 ×10³/µL	4.0–10.6 ×10³/µL
	Hemoglobin	16.3 g/dL	12.0–14.1 g/dL
	Hematocrit	51.1 %	35.6–43.0 %
	Platelet count	360 ×10 ³/µL	154–407 ×10³/µL
	PT (INR)	1.00	0.8–1.2
	aPTT	36.1 sec	27–45 sec
	AST	32 U/L	5–40 U/L
	ALT	51 U/L	3–40 U/L
	BUN	9.9 mg/dL	5–23 mg/dL
	Creatinine	0.36 mg/dL	0.6–1.1 mg/dL
	Sodium	139.5 mEq/L	136–150 mEq/L
	Potassium	4.37 mEq/L	3.5–5.3 mEq/L
	Albumin	4.2 g/dL	3.5–5 g/dL
	hsTroponin-I	5 ng/L	0–17.5 ng/L
	NT-proBNP (Pro-BNP)	4.73 pg/mL	0–125 pg/mL
	Fasting glucose	123 mg/dL	70–120 mg/dL
	HbA1c (NGSP)	7.6 %	4.3–6.0 %
ABGA	pH	7.393	7.35–7.45
	PaCO₂	43.0 mmHg	35–45 mmHg
	PaO₂	77.4 mmHg	75–100 mmHg
	HCO₃⁻	25.6 mmol/L	20–29 mmol/L
	Base excess	0.6 mmol/L	—
	SaO₂	95.3 %	95–98 %
ECG	Rhythm/Rate	Sinus tachycardia, 109 bpm	—
	QTc	463 ms	—
Spirometry (pre/post BD)	FVC	1.91 L (56% pred) → 1.84 L (54% pred)	—
	FEV1	1.72 L (70% pred) → 1.69 L (69% pred)	—
	FEV1/FVC	90% → 92%	—
	FEF25–75	3.23 L/s (107% pred) → 3.49 L/s (116% pred)	—
Exercise treadmill test (Bruce)	Exercise duration / Max METS	2 min 12 sec / 4.5 METS	—
	HR (rest → max)	115 → 160 bpm (83% predicted max)	—
	BP (rest → max)	166/106 → 194/52 mmHg	—
	Reason for stopping	Dyspnea, leg pain	—
PSG	AHI	111.9 events/hr	—
	RDI	123.4 events/hr	—
	Baseline SpO₂	80%	—
	Lowest SpO₂	58%	—
	REM/TST	0.0%	—
	Respiratory arousal index	96.4 /hr	—

**Figure 1:**
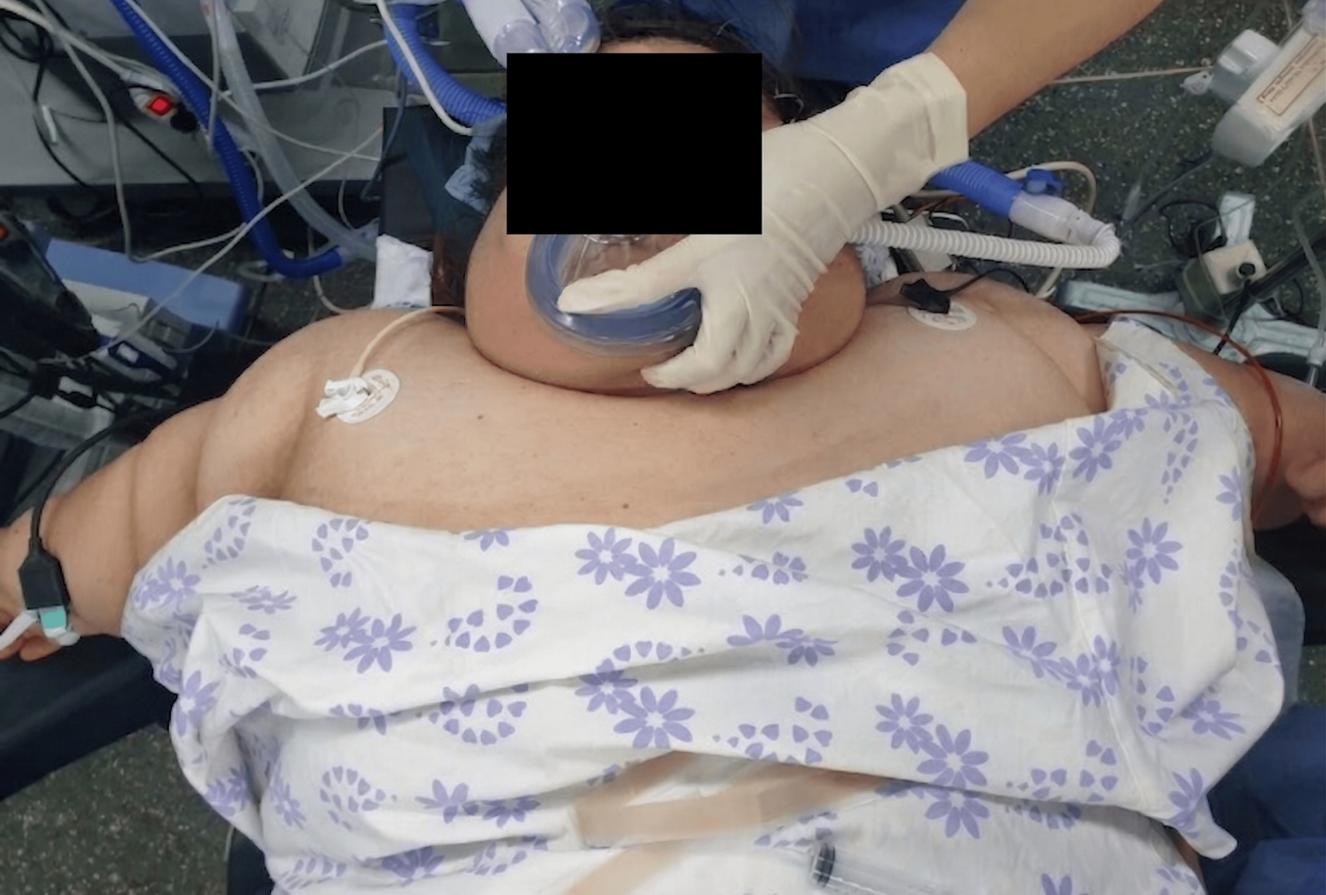
Pre-intubation airway preparation before induction of anesthesia The photograph illustrates the pre-intubation airway setup, including high-flow nasal oxygen (HFNO), and the patient’s short neck and marked cervicothoracic soft tissue burden, which were relevant to difficult airway management. HFNO, high-flow nasal oxygen.

To prepare for a difficult airway, high-flow nasal oxygen (HFNO), a McGRATH™ MAC video laryngoscope (standard blade, size 3; X-blade, size 3), a flexible bronchoscope, and sugammadex were made available. After arrival in the operating room, standard monitoring was applied, and HFNO (60 L/min, FiO₂ 1.0) was initiated before induction. Figure 1 illustrates the pre-intubation airway setup, including HFNO, and the patient’s short neck and marked cervicothoracic soft tissue burden.

A 22-gauge catheter was placed in the right radial artery under ultrasound guidance. Anesthesia was induced with propofol 160 mg, rocuronium 50 mg, and a continuous remifentanil infusion. The initial intubation attempt using the videolaryngoscope with a standard blade (size 3) showed a modified Cormack-Lehane grade III view and was unsuccessful. A ramped position was attempted; however, optimal alignment of the external auditory meatus with the sternal notch was unattainable due to her disproportionately short torso, and excessive neck extension was considered potentially hazardous. A second attempt using the X-blade (size 3) and a styletted endotracheal tube shaped in a hockey-stick configuration was successful. From the first laryngoscopic attempt to successful tracheal intubation with the X-blade, airway management required approximately 4 minutes. With HFNO in place, no clinically significant decrease in SpO₂ occurred during airway management. A 7.0-mm cuffed oral endotracheal tube was secured at 18 cm.

Anesthesia was maintained with desflurane (6 vol%, FiO₂ 0.5-0.6) and a continuous remifentanil infusion. Additional rocuronium was administered based on quantitative electromyography neuromuscular monitoring (TwitchView®), guided by train-of-four (TOF) measurements. The bispectral index (BIS) was maintained between 40 and 60. Mechanical ventilation was set to a tidal volume of 300 mL, respiratory rate of 12-15 breaths/min, and positive end-expiratory pressure of 7 cmH₂O; peak and plateau airway pressures were 29 and 28 cmH₂O, respectively. Ventilatory parameters remained stable following the initiation of pneumoperitoneum. At the end of surgery, a Train-of-Four (TOF) count of 2 was confirmed, and sugammadex 200 mg was administered intravenously. After confirming a TOF ratio ≥0.9, the trachea was extubated. On arrival to the postanesthesia care unit, the patient was hemodynamically stable and fully awake.

**Figure 2:**
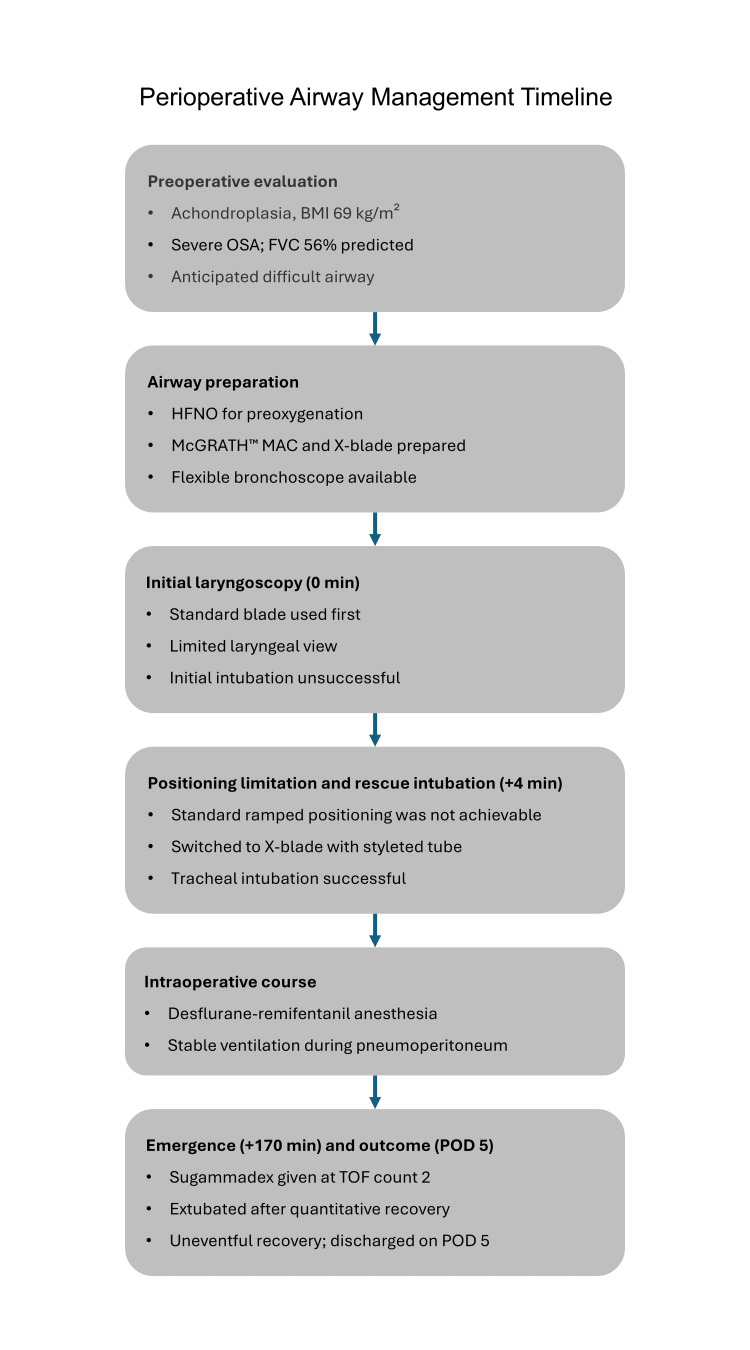
Perioperative airway management timeline Perioperative airway management timeline in the present case. The figure summarizes the preoperative findings, airway preparation, initial unsuccessful laryngoscopy, successful rescue intubation with an X-blade and styleted tube, stable intraoperative course, emergence after quantitative neuromuscular monitoring and sugammadex reversal, and discharge on postoperative day five after an uneventful recovery. HFNO: high-flow nasal oxygen; OSA: obstructive sleep apnea; FVC: forced vital capacity; TOF: train-of-four; POD: postoperative day.

Postoperatively, she recovered without notable complications, was managed on the general ward, and was discharged on postoperative day five without adverse events. The major perioperative airway management events are summarized in Figure 2.

## Discussion

This case describes the anesthetic management of laparoscopic sleeve gastrectomy in a patient with achondroplasia who had previously undergone limb-lengthening surgery and presented with morbid obesity and severe obstructive sleep apnea. Because limb-lengthening procedures are typically confined to the skeletal structures of the lower extremities, the anatomic characteristics of the upper body structures, such as thoracic cage size, airway anatomy, cervical spine structure, and spinal canal dimensions, would not be expected to change proportionally. Thus, reliance on the measured height alone may be misleading when anesthetic risk is assessed. In this patient, upper-airway and thoracic features may have more closely reflected the estimated pre-lengthening height (approximately 125 cm) than the final standing height. Height-based assessment alone may therefore underestimate true achondroplasia-related anesthetic risk after limb lengthening.

This disproportion was also reflected in the pulmonary evaluation. The patient’s FVC was 56% of predicted, suggesting a possible restrictive ventilatory pattern. However, the predicted values were calculated using a standing height of 142.3 cm, and in achondroplasia complicated by limb lengthening, the usual relationship between standing height and thoracic dimensions may not apply. Height-based predicted values may therefore misrepresent the degree of ventilatory impairment [3]. Despite this limitation, the overall findings still suggested markedly reduced respiratory reserve and increased perioperative respiratory risk.

This upper-lower body disproportion also affected positioning for airway management. In obese patients, the ramped position is commonly used to align the external auditory meatus with the sternal notch and thereby improve the laryngoscopic view [6,7]. In the present patient, however, the trunk remained disproportionately short, with a markedly shortened thorax, so elevation beneath the shoulders alone was insufficient to achieve the desired alignment. More aggressive positioning could instead provoke cervical hyperextension, which should be avoided in achondroplasia because of the risk of foramen magnum stenosis and cervical instability during tracheal intubation [3]. This case illustrates that standard ramped positioning may not be directly applicable when disproportionate trunk shortening persists. This interpretation is also consistent with current achondroplasia anesthesia recommendations [3], which emphasize individualized airway planning and strict avoidance of unnecessary cervical hyperextension in patients at risk of craniocervical compression.

Before induction, concern focused not only on the technical difficulty of airway management but also on the risk of rapid desaturation during airway manipulation. Baseline SpO₂ of 80%, a nocturnal SpO₂ nadir of 58%, an AHI of 111.9, morbid obesity, and findings suggestive of a restrictive ventilatory pattern collectively indicated markedly reduced oxygen reserve and an anticipated shortening of safe apnea time. HFNO was therefore used as a core safety strategy rather than a simple precaution. By continuously delivering heated, humidified high-flow oxygen to the upper airway, HFNO can enhance preoxygenation and provide apneic oxygenation, which may prolong safe apnea time [8,9]. In this case, despite additional time for airway management because of suboptimal positioning and the need to change laryngoscopes, no clinically significant decrease in SpO₂ occurred, supporting the value of this strategy.

In the previously reported bariatric surgery cases in patients with achondroplasia that we identified, airway management was achieved using flexible bronchoscopy, including awake techniques, as either a primary or rescue approach for tracheal intubation [4,5]. In contrast, the present case suggests that a hyperangulated videolaryngoscope may also be an effective alternative when optimal positioning cannot be achieved, and flexible bronchoscopy is available as backup. Hyperangulated videolaryngoscopes have been associated with improved glottic visualization [10], and our experience suggests that this approach may be particularly useful when positioning is suboptimal and unnecessary cervical manipulation should be minimized.

Drug dosing was another challenge. In morbid obesity, the volume of distribution and clearance of anesthetic drugs vary according to the specific drug; therefore, uniform dosing based on actual body weight may increase the risk of overdosing, whereas reliance on ideal body weight alone may be inadequate for induction or tracheal intubation [11]. In this patient, the unusual body proportions made it even less likely that a single weight scalar would be appropriate for all drugs [12]. We therefore adopted a drug-specific strategy, using lean body weight (LBW) as a conservative starting point when appropriate and titrating to effect with clinical and quantitative monitoring.

Propofol was titrated using a scalar close to LBW, consistent with reports that LBW approximates the induction requirement while limiting overdosing [13]. Remifentanil was also started on an LBW-based regimen, as its pharmacokinetic parameters correlate more closely with LBW than with actual body weight (ABW) in obesity [14]. Rocuronium, a hydrophilic drug, may have a prolonged duration when dosed by ABW in obese patients; accordingly, conservative initial dosing with subsequent supplementation under quantitative neuromuscular monitoring was considered more appropriate than routine ABW-based dosing [15,16]. These considerations support drug-specific dosing rather than reliance on a single scalar.

Safe emergence was also a major concern. In patients with extremely severe obstructive sleep apnea, residual anesthetic effect and neuromuscular block may delay recovery of upper-airway muscle function and increase the risk of postoperative airway obstruction and respiratory depression [17]. To facilitate emergence, desflurane was selected for maintenance because its low blood/gas partition coefficient permits rapid washout, and anesthetic depth was titrated with BIS (target 40-60) to avoid excessive anesthesia.

Quantitative electromyography-based neuromuscular monitoring was applied throughout, and sugammadex was used for reversal at the end of surgery [18]. Although ABW-based sugammadex dosing has been associated with faster recovery than ideal body weight-based dosing in morbidly obese patients [19], the present patient’s body composition and distribution volume were difficult to predict using a single scalar. We therefore prioritized objective confirmation of recovery rather than theoretical precision of the nominal dose and proceeded with extubation only after verifying a train-of-four ratio ≥0.9. In this setting, quantitative confirmation of recovery functioned as the key safety safeguard against uncertainty in weight-scalar-based dosing.

## Conclusions

This case suggests that in achondroplasia, after limb-lengthening, standing height may not adequately reflect airway or thoracic risk. Safe anesthetic management requires attention to body proportions rather than measured height alone, advanced planning for difficult airways and oxygenation, drug-specific dosing with monitoring, and objective confirmation of complete neuromuscular recovery. Because these observations derive from a single case, further clinical experience and additional reports are needed to clarify their broader applicability.
